# The Roles of Some Scorpions, *Hemiscorpius lepturus* and *Androctonus crassicauda*, in a Scorpionism Focus in Ramhormorz, Southwestern Iran

**DOI:** 10.1673/031.013.8901

**Published:** 2013-09-22

**Authors:** Alireza Mohseni, Babak Vazirianzadeh, Mohsen Hossienzadeh, Maryam Salehcheh, Azra Moradi, Seyed Abbas Moravvej

**Affiliations:** 1 Department of Hematology, School of Paramedicine, Ahvaz Jundishapur University of Medical Sciences, Ahwaz, Iran, and Department of Laboratory Sciences, Faculty of Paramedicine, Mazandaran University of Medical Sciences, Sari, Iran; 2 Department of Medical Entomology, School of Health, and Infectious and Tropical Diseases Research Center, Ahvaz Jundishapur University of Medical Sciences, Ahvaz, Iran; 3 Department of Pediatrics, College of Medicine, Jundishapur University of Medical Sciences, Ahvaz, Iran; 4 Department of Toxicology, School of Pharmacy, Ahvaz Jundishapur University of Medical Sciences, Ahwaz, Iran; 5 Imam Khomeini (RH) Hospital, Ramhormoz, Ahvaz Jundishapur University of Medical Sciences, Ahwaz, Iran; 6 Department of Entomology, College of Agriculture, Chamran University, Ahvaz, Iran

**Keywords:** biochemical analysis data, epidemiological, hematological analysis data, scorpion sting, urine analysis data

## Abstract

Scorpion stings are a common and important health problem in Iran, particularly in south and southwestern Iran, including the province of Khuzestan. In the area of Khuzestan near the city of Ramhormoz, *Hemiscorpius lepturus* (Scorpionida: Hemiscorpioiidae) and *Androctonus crassicauda* (Buthidae) are present. Ramhormoz is in southwestern Iran and is one of the most important foci of the scorpion sting problem. The current study was carried out to gain both epidemiological and medical information about scorpion stings in and around the city of Ramhormoz. In total, 179 people who were admitted to the Emergency Department of Ramhormoz Imam Khomeini Hospital during 2008 and 2009 after being stung by scorpions were monitored. Epidemiological and medical parameters including sex of the victim; the part of the body stung; the month when stung; the biochemical parameters comprising blood sugar (BS), blood urea nitrogen (BUN), and creatinine (CR); hematological parameters including white blood cells (WBC), count blood cells (CBC), red blood cells (RBC), hemoglobin (Hb), hematocrit (HCT), platelet (PLT); and urine analysis including hemoglobinuria were recorded. The current study showed that most of the victims were stung by *H. lepturus*, while very few were stung by *A. crassicaud*, but in over half of the cases the species was not known. Stings were most common from May to Aguust. 73% of the victims were female. The limbs were the part of the body most likely to be stung. Hemogobinuria was very common in *H. lepturus* victims.

## Introduction

Scorpion stings are a common and important health problem in Iran, particularly in south and southwestern Iran. From 2001 to 2005, 192,351 cases were reported, in which 104 cases resulted in death (Deghani 2003; Deghani et al. 2004; Deghani and Valaie 2005; Azhang and Moghisi 2006). Most of the cases, as well as those resulting in mortality, occurred in Khuzestan, a southwest province of Iran (Azhang and Moghisi 2006), and scorpionism generally is endemic in Khuzestan (Pipelzadeh et al. 2007).

The Iranian scorpion (Scorpionida) fauna consists of over 44 named species from 23 genera in two families, Buthidae and Scorpionidae. However, *Hemiscorpius lepturus*, belongs to the Hemiscorpiidae family, and is the most medically important scorpion in Iran (Farzanpey 1987; Lorenço 2001; Lowe 2010).

Species of *Hemiscorpius lepturus, Androctonus crassicauda* (Buthidae), and *Mesobuthus eupeus* are the main species responsible for stings in this area (Chitnis et al. 1993; Afzali and Pezeshki 1998; Pipelzadeh et al. 2007), but *H lepturus* is the most venomous of all types of scorpions in the region, and contributes to 95% of all mortalities in scorpionstung patients. *H. lepturus* has been variously reported as responsible for 10–15% of scorpion stings in Khuzestan (Radmanesh 1990a, b, 1998; Shahbazzadeh et al. 2007; Zare Mirakabbadi et al. 2007), or 30% of scorpion stings overall in Khuzestan (Vazirianzadeh and Samie 2005; Vazirianzadeh et al. 2008). This scorpion species is distributed in Iran, Iraq, Pakistan, and Yemen (Lorenço 2001); however, Lowe (2010) reported two new species of the genus *Hemiscorpius, H. falcifer* and *H. flagelliraptor*, from Oman.

*H. lepturus* is well known for having a potent cytotoxic venom that causes cutaneous necrosis, deep and necrotic ulcers, psychological problems, ankylosis of the joints, and severe systemic pathology leading to death, severe and fatal haemolysis, secondary renal failure, and fatal failure of the kidney (Afzali and Pezeshki 1998; Radmanesh 1998; Pipelzadeh et al. 2006, 2007; Jalali 2010; Lowe 2010). *H. lepturus* is the only scorpion with related cutaneous findings in Iran (Radmanesh 1990b). However, Monod and Lourenço (2005) have proposed a new hypothesis regarding the genus of *Hemiscorpius* in Iran that are morphologically very close to each other and difficult to distinguish for a non-specialist; if so, *H. lepturus* is probably not the only species responsible for all envenomation in Iran.

*A. crassicauda* is the second most dangerous scorpion in Iran (Radmanesh 1990a, b, 1998; Pipelzadeh et al. 2007), and has a large geographical distribution in both the world and Iran. Vazirianzadeh et al. (2008) reported that 27% of scorpion stings in April-September 2007 were caused by *A. crassicauda*. Dehgani et al. (2009) reported this rate as 29% in Khuzestan. The toxin can cause severe pain, autonomic, CNS, and muscle function disturbances, and death (Radmanesh 1990a).

Like *Hemiscorpius*, there are similar views in terms of there being more than one species of *Androctonus* genus in Iran. There are two other species of this genus other than *A. crassicauda* in Iran, namely *A. baluchicus* in east Iran and *A. amoreuxi* in southwest Iran (Farzanpey 1987; Vazirianzadeh 1990; Mirshamsi et al. 2011).

Both *H. lepturus* and *A. crassicauda* are present in the area of Khuzestan, including Ramhormoz city. The Ramhormoz area,which is in east Khuzestan, is one of the most important foci of the scorpion sting problem, particularly *H. lepturus* stings, from the point of epidemiology and the geographic and local scorpion distribution, according to the reports of local health authoritis of Khuzestan, and is followed by *A. crassicauda* (Radmanesh 1990a, b, 1998; Vazirianzadeh and Samie 2005; Pipelzadeh et al. 2007; Vazirianzadeh et al. 2008).

This retrospective study was carried out to investigate and evaluate the roles of *H. lepturus* and *A. crassicauda* stings from the points of the epidemiology and its effects on biochemical, hematological, and urine lab parameters among the scorpion stung people of this region in order to provide guidance to determine the best treatment of scorpion stings. However, as mentioned, there are several species of both genera in Khuzestan that affect the clinical and paraclinical results of scorpion stings in the patients.

## Materials and Methods

Ramhormoz, at 31° 17′ 0″ N, 49° 36′ 0″ E, in Khuzestan province in southwestern Iran is an ancient city with a rural and tribal social structure.

This research was a descriptive retrospective study. The data of the present research came from files of outpatient or hospitalized persons referred to the health center and hospitals in the city of Ramhormoz during one year (22 March 2008–21 March 2009).

In the current study, the data of scorpion stings were studied from the points of epidemiological and medical lab parameters. The data included: the sex of the scorpion-stung victim; the body part stung; the month of the scorpion sting; the biochemical parameters blood sugar (BS), blood urea nitrogen (BUN), and creatinine (CR); the hematological parameters white blood cells (WBC), count blood cells (CBC), red blood cells (RBC), hemoglobin (Hb), hematocrit (HCT), and platelet (PLT); and urinalysis hemoglobinuria.

The frequencies of the epidemiological and medical lab parameters were converted to the percentage rank. A *t*-test was used to compare the results. A *p*-value of < 0.05 was used for the level of significance.

## Results

In total, 179 files for the stung people admitted to the emergency department of Ramhormoz Imam Khomeini Hospital were monitored during 2008 and 2009. In 83 of the cases, the scorpion species responsible for the sting was recorded as being either *H. lepturus* or *A. crassicauda* (77 and 6 cases, respectively). The scorpion species responsible for the remaining cases were unknown. 131 of the patients were female (73 %) (Table 1).

Table 2 shows the frequency of stings on each body part related to the species of scorpion. It shows that 46.88 % and 66.67 % of stings were in the foot by *H. lepturus* and *A. crassicauda*, respectively. These results came from 179 patients.

Table 3 shows the frequency of stings in each month. Stings happened most frequently from May through August.

Biochemical blood test results are shown in the Table 4, and include BS, BUN, and CR data. These data were classified based on the species. Means ± SD of BS, BUN, and CR were 117 ± 41 mg/dL, 14 ± 5 mg/dL, and 0.7 ± 0.2 mg/dL for *H. lepturus* and 140 ± 63 mg/dL, 13 ± 3mg/dL, and 0.6 ± 0.1 mg/dL for *A. crassicauda*. The other data belong to unidentified species.

The results of hematological parameters, including WBC, RBC, HB, HCT, PLT, PTT, and PT, are shown in Tables 5–8 according to the scorpion species. All of the above mentioned parameters were in the normal range.

The results of the hemoglobinuria test, which are presented in Table 9, show that the most severe hemoglobinuria, + 4, occurred in the victims of *H. lepturus*, which constituted 7% of this group and 4% of the total.

## Discussion

### Epidemiological data

Scorpionism studies in Iran have been restricted to Khuzestan province. Data on scorpion stings in Ramhormorz, in the eastern region of Khuzestan, showed that the two most important scorpion species in this area are *H. lepturus* and *A. crassicauda*, in terms of the percentages of scorpion stings. There is very little specific data regarding the other species, except that *M. eupeus* is counted as the third most important scorpion in Khuzestan in terms of percentage of stings.

The results of the present study showed that most of the patients with scorpion stings were female (73%). This rate is in accordance with the results of Vazirianzadeh and Samie (2005) for Khuzestan. However, it is not consistent with the results of Dehgani et al. (2010) in Kashan; they reported that the percentage of stings were about the same for males and females. The results of the present study were also not in accordance with the results of AlSadoon and Jarrar (2003) and Jarrar and AlRowaily (2008) in Saudi Arabia. This could be due to two reasons: first, different cultural factors, and second, more people are at risk of scorpionism in Ramhormoz than the other areas studied.

Vazirianzadeh et al. (2008) reported that most scorpion-sting victims were housewives (41%) out of 997 studied patients in the Province of Khuzestan. This result is in accordance with the results of the present study, with females being dominant among the patients. There is a sociocultural practice that the women help with post-harvest treatments of crops, such as making the bundles of vegetables or packaging the other crops of the area. Many scorpion stings occur during this post-harvest work. Vazirianzadeh et al. (2008) also reported that 91% of scorpion stings happened at home, including 92% of *A. crassicauda* and 98% of *H. lepturus* stings. Both species are non-drilling scorpions, and can hide themselves everywhere. Most scorpion sting cases are reported from the Ramhormoz region. This region has the most scorpions in the province (http://pezeshkan.ir/view.asp.2008),therefore the local people are at high risk of being stung by a scorpion.

The results of this research revealed that patients were stung on the legs more than the other parts of body. Both species, *H. lepturus* and *A. crassicauda*, targeted the limbs with frequencies of 72% and 67%, respectively, compared to the other parts of the body. This result agrees with studies of Al-Sadoon and Jarrar (2003) and Jarrar and Al-Rowaily (2008) in Saudi Arabia. The total data, regardless of species, followed a similar trend in terms of sites of scorpion stings on the bodies of humans (Table 2). Therefore, suitably covering both limbs would help to prevent scorpion stings.

The current study revealed that the season with the most scorpion-sting cases was summer (44%). This result is in accordance with the studies of Chitnis et al. (1993), Vazirianzadeh and Samie (2005), and Vazirianzadeh et al. (2008) in Iran; Al-Sadoon and Jarrar (2003) and Jarrar and Al-Rowaily (2008) in Saudi Arabia; and Ozkan and Kat (2005) and Ozkan et al. (2006) in Turkey, who reported that 50–93% of scorpion sting cases occurred in the summer. Differences in these results were presumably due to the variation of geography, climate, and species distribution. However, the results of the current study show that the *H. lepturus* was most active in May (22% of annual stings), and *A. crassicauda* was most active in August (33%). These results are confirmed by the results of Vazirianzadeh and Samie (2005), who reported that *H. lepturus* was most active in May (8%), and *A. crassicauda* was most active in August (10%). Therefore, the more important scorpionism in the mild temperate seasons is due to *H. lepturus*, and to *A. crassicauda* during warmer seasons. These results, however, are not in accordance with the results of Pipelzadeh et al. (2007) regarding *H. lepturus* activity. They reported July as the month with the most *H. lepturus* stings. However, this report concerned the mean activity of scorpions in Khuzestan as a whole, and the presentstudy data were related to the Ramhormoz region only.

### Biochemical data

In the current study, biochemical results including rates of BS, BUN, and CR in the stung people were in the normal rates, and there was not any significant difference between those rates and normal values (means ± SD, Table 4). These results are similar to the results of Vazirianzadeh and Samie (2005). Consequently, BS, BUN, and CR levels are not areas of major concern in regards to scorpionism in this region, according to the early results of the biochemical tests of scorpionstung patients. However, these levels may require further study, because secondary renal failures due to scorpion stings have been reported by several authors in Khuzestan. Increasing BUN and CR levels in sting victims are good markers of renal failure following a scorpion sting (Chadha and Leviav 1979; Malhotra et al. 1978; Radmanesh 1990a, b).

The rates of BS increased significantly in experimental rabbits in the case of *H. lepturus* stings (Pipelzadeh et al. 2007; Zare Mirakabbadi et al. 2007). The differences of this result from the results of the human study may be due to injection of lethal doses in the rabbit studies.

All the above comparative issues were discussed on the basis of the means of the biochemical parameters. The maximum levels of BS in the cases of *H. lepturus, A. crassicauda*, and other scorpion species victims were recorded as 270 mg/dL, 220 mg/dL, and 245 mg/dL, respectively. The maximum BUN levels were recorded as 29 mg/dL, 16 mg/dL, and 31 mg/dL, respectively. These results suggest that the venom of scorpions in Ramhormoz increased the levels of BS in some of victims. This is in contrast to the rate of BUN, which increased in some victims by levels of 29 and 31 mg/dL in the cases of *H. lepturus* and unidentified species of scorpions, in which they were higher than normal levels. In the case of *A. crassicauda*, this value was in the normal rate in the current study (as the maximum recorded levels). Therefore, the levels of BS and BUN of the blood may be used as factors signifying scorpionism by *H. lepturus* in Ramhormorz. In the case of *A. crassicauda* stings, only BS level can be used as a factor signifying scorpionism in Ramhormorz. However, the alteration in levels of BS in the blood tests must be interpreted before treatment with dextrose injection to the patients, which is a common treatment in the terms of scorpion sting in Khuzestan, especially for *H. lepturus* stings.

Finally, based on the results of this study and the views of several other authors, the levels of BS, BUN, and CR in scorpion victims could be considered as paraclinical markers. However, the mentioned factors are affected by several factors, such as age, overall health of the victim, the site of the scorpion sting on the body of the victim, and the age of the scorpion. These parameters in the Ramhormoz area must be considered regarding the mean ages ± SD of 35 ± 15.49 and 26.52 ± 17.18 years old, as an important factor among the stung scorpion victims, for *A. crassicauda* and *H. lepturus*, respectively (Radmanesh 1990 a, b; Valavi and Alemzadeh Ansari 2008).

### Hematological and urine analysis data

The results of CBC measurements showed no significant increasing in WBC in the threegroups of scorpion victims (*p* > 0.05). However, this result is not in accordance with the results obtained by Chitnis et al. (1993), who reported an elevated WBC in the majority of the patients that died following scorpion stings. The results of the current study are in accordance with the studies of Vazirianzadeh et al. (2008). However, in the current study, leucocytosis was seen in 42% of *H. lepturus* victims, but it was not seen in the *A. crassicauda* victims. It is presumed that inflammation reactions caused leucocytosis in the *H. lepturus* cases. This result is consistent with the results of Emam et al. (2011), in which there was not any significant difference between *H. lepturus* victims and natural standards in the MCV, MCH, and MCHC parameters, in the Hendijan district, south of Khuzestan.

In the present study, the means of RBC counts in the *H. lepturus, A. crassicauda*, and unknown-scorpion victims were in the normal ranges. However, the minimum recorded RBC counts, with a value of 1.26 × 10^12^/L, in the *H. lepturus-stung* patients suggested that there was a haemolysis in these people. This value was much lower than in the *A. crassicauda* stung patients, who had the value of 3.69 × 10^12^. The RBC-count results in the present study among *H. lepturus* vicitms with the minimum records of RBC counts, 1.26 × 10^12^/L, are more or less in agreement with Salimian et al. (2002), Dehghani et al. (2004), Pipelzadeh et al. (2006, 2007), Mirakabbadi et al. (2007), and Jalali et al. (2010), who have reported RBC reduction in several experimental animals. The hemolysis effect of *H. lepturus* venom has also been documented using laboratory findings in humans. Farzanpey (1994) mentioned the hemolytic symptoms of *H. lepturus* venom in some victims. This result showed that the venom of *H*.*lepturus* had a greater effect on erythrocyte hemolysis than the venom of *A. crassicauda*.

Hb and HCT values were in the normal range for all of the scorpion victims in the present study. However, in the some of the victims, the rates of Hb and erythrocyte counts were lower than normal. This reduction was due to erythrocyte hemolysis. This result agrees with the results of Vazirianzadeh and Samie (2005) in Khuzestan. These results are also similar to the results of Emam et al. (2008) and Emam et al. (2011) in Hendijan and Khuzestan, respectively, who found reductions in the amounts of both parameters, but no statistical difference was observed among the *H. lepturus*stung patients from the standard amounts. Pipelzadeh et al. (2007) reported a rapid drop in the level of HCT with a severe hemolysis among people referred to hospital emergency care. Emam et al. (2008) reported similar results, including reduction in RBC and HCT amounts among the people stung by *H. lepturus* in Khuzestan province.

PTT, PT, and PLT (× 10^9^/L) counts were in the normal range in all scorpion-victim cases. This normalcy shows that these factors were not important indices in scorpion sting cases. This result is not in agreement with the results obtained by Emam et al. (2011) and Murthy and Zara (2001). They reported that PTT, PT, Hb, RBC, and PLT (× 10^9^/L) counts were considered as important indices in *H. lepturus* victims.

The results of the present study showed that there was severe hemogobinuria in 95% of *H. lepturus* victims with greater than +1; however, this rate also occurred in 17% of *A. crassicauda* victims. This difference occurred because erythrocyte hemolysis was more severe in the victims of *H. lepturus* in Ramhormoz. The hemogobinuria decreased with time. Therefore, monitoring hemogobinuria is considered as an essential parameter in the recovery period of *H. lepturus* victims compared to *A. crassicauda* victims. This conclusion is in accordance with other researchers (Radmanesh 1990a, b, 1998; Murthy and Zara 2001; Shahbazzadeh et al. 2007; Vazirianzadeh et al. 2008; Emam et al. (2011). However, Radmanesh (1990a, b, 1998), Vazirianzadeh et al. (2008), and Emam et al. (2011) reported hemogobinuria in both *H. lepturus* and *A. crassicauda* victims, the majority of which were *H. lepturus* stung victims. Afzali and Pezeshki (1998) reported that renal failure due to a *H. lepturus* sting is a secondary phenomenon, and the venom of this species is not nephrotoxic. They have also explained that hemoglobinuria is the most important sign of a *H. lepturus* sting, and can be followed by renal failure. This conclusion is in agreememnt with the results of the present study.

All the mentioned lab results regarding the hematological and urine data should be analyzed and interpreted together as multifactorial data. The reduction of RBC, hemogobinuria, and produced anemia must be considered as parallel and accompanied together. Valavi and Alamzadeh Ansari (2008) explained a combination of microangiopatic hemolytic anemia, thrombocytopenia, and acute renal failure in their study following a *H. lepturus* sting, leading to a diagnosis of hemolytic uremic syndrome.

Finally, there are valuable reasons to further study and interpret the differences between our results and the results of other similar studies regarding hematological and biochemical data. The most important reasons for these differences are related to using different geographical areas and methods of studies.

The difference in geographical area is considered to be the major reason why the present study obtained different results than the Emam et al. (2011) study in Hendijan. Hendijan is in the south of Khuzestan while Ramhormoz is in the east. With respect to the Monod and Lourenço study (2005), the hypothesis that there are different species of *Hemiscorpius* or subspecies of *H lepturus* is raised up by the authors of the present study. Similar hypotheses may be considered regarding *A. crassicauda* based on Farzanpey (1987), Vazirianzadeh (1990) and Mirshamsi et al. (2011) studies. *A. amorexi* is present in the field of Khuzestan.

The reason the results of the present study differed from the results of the Emam et al (2008) study is that different geographical areas and methods were used. Emam et al. performed their study in the Ahwaz area, in the central part of the province, and their data came from Razi hospital, a central hospital for scorpion-sting victims to be taken to in the province, meaning their study was based on the average data of Khuzestan.

The authors of the present study suggest the application of alkaline diuresis to regulate alkalosis of the urine as a medical approach against renal failure due to the hemolysis effects of *H. lepturus*, and antivenom treatments to reduce neurotoxic effects of *A. crassicauda* venom (Farzanpey 1987; Radmanesh 1998). This antivenom is a 5 mL polyvalent ampoule against six species including *H. lepturus, A. crassicauda, Mesobuthus eupeus, Odonthobothus doriae, Hottentotta saulcyi*, and *Hottentotta schach*. It is made at the Razi Research Vaccine and Serum Institute, Iran.

The present study was carried out in one area, including both *H. lepturus* and *A. crassicauda*, with an emphasis on paraclinical data. Thedifference between the results of the current study and the other similar studies, especially regarding *H. lepturus*, is due to the possibility of the existence of different subspecies of *H. lepturus* and *A. crassicauda*, or different species of *Hemiscorpius* and *Androctonus* genera in Khuzestan (Farzanpey 1987; Vazirianzadeh 1990; Monod and Lourenco 2005; Mirshamsi et al. 2011). These different species or subspecies may be the cause of different epidemiological, biochemical, hematological, and urine analysis data among the scorpionsting victims in the different cases and areas; however, there are no published data regarding the presence of different subspecies of either species, and further study is needed in the different areas of Khuzestan. Other reasons could also explain the differences in results, such as different areas of study and different methods used.

Urine analysis data are considered to be the most important in the follow-up of scorpion victims and during their recovery period. Because *H. lepturus* stings are more dangerous than *A. crassicauda* stings, identification of the species involved is very helpful. Gathering these paraclinical data should be considered in the warmer months for *A. crassicauda* victims and in the temperate months of spring for *H. lepturus* victims.

**Table 1.**
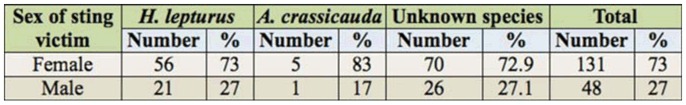
Number and percentage of scorpion stings according to the gender of the sting victims and the species of scorpion.

**Table 2.**
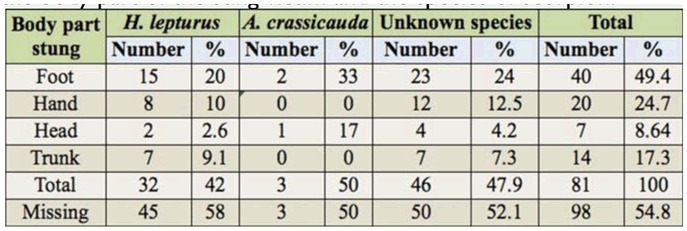
Number and percentage of scorpion stings according to the body part of the sting victim and the species of scorpion.

**Table 3.**
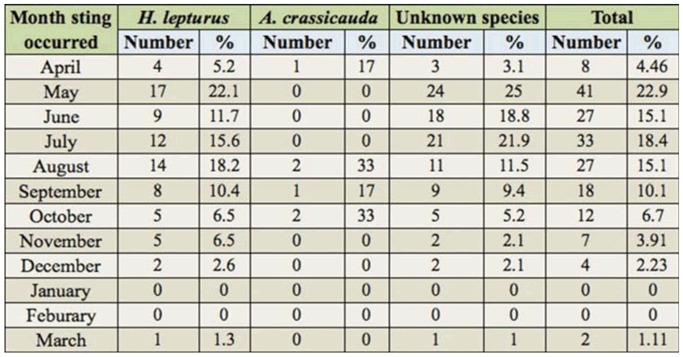
Number and percentage of scorpion stings according to the month the sting occurred and the species of scorpions.

**Table 4.**
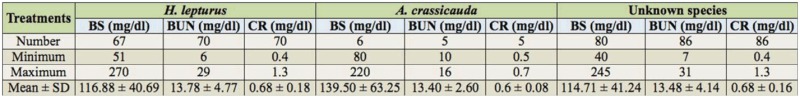
Blood test results according to scorpion.

**Table 5.**
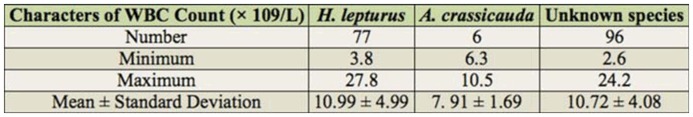
Characters of WBC count in patients stung by scorpions in Ramhormoz in 2008–2009.

**Table 6.**
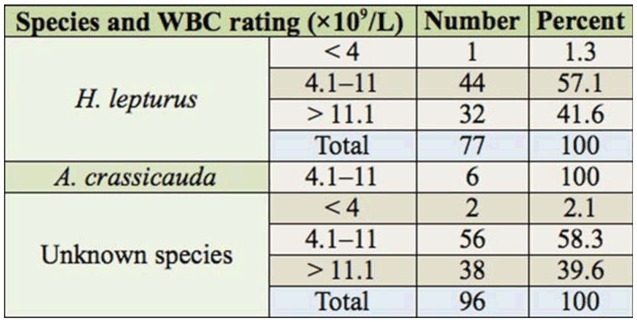
Rating of WBC count in the patients stung by scorpions in Ramhormoz in 2008–2009.

**Table 7.**
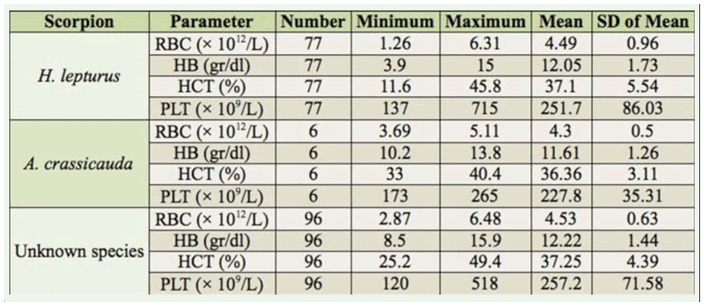
Hematologic parameters in patients stung by scorpions in Ramhormoz in 2008–2009.

**Table 8.**
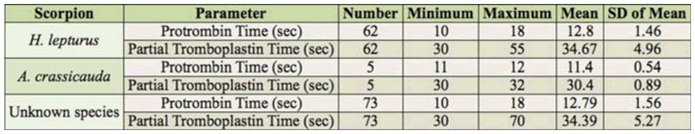
Rates of protrombin time and partial tromboplastin time in patients stung by scorpions in Ramhormoz in 2008–2009.

**Table 9.**
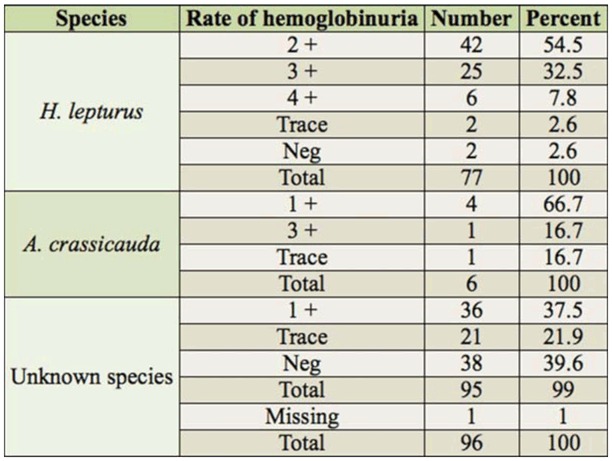
Rates of hemoglobinuria in patients stung by scorpions in Ramhormoz in 2008–2009.

## Abbreviations:

BUN,: blood urea nitrogen
CBC,: count blood cells
CR,: creatinine
Hb,: hemoglobin
HCT,: hematocrit
PLT,: platelet
RBC,: red blood cells
WBC,: white blood cells
